# Elucidating the Efficacy of the Bacille Calmette–Guérin Vaccination in Conjunction with First Line Antibiotics and Liposomal Glutathione

**DOI:** 10.3390/jcm8101556

**Published:** 2019-09-27

**Authors:** Rachel Abrahem, Ruoqiong Cao, Brittanie Robinson, Shalok Munjal, Thomas Cho, Kimberly To, David Ashley, Joshua Hernandez, Timothy Nguyen, Garrett Teskey, Vishwanath Venketaraman

**Affiliations:** 1 Graduate College of Biomedical Sciences, Western University of Health Sciences, Pomona, CA 91766-1854, USA; rachel.abrahem@westernu.edu (R.A.); kimberly.to@westernu.edu (K.T.); david.ashley@westernu.edu (D.A.); joshua.hernandez@westernu.edu (J.H.); 2 College of Osteopathic Medicine of the Pacific, Western University of Health Sciences, Pomona, CA 91766-1854, USA; brittanie.robinson@westernu.edu (B.R.); shalok.munjal@westernu.edu (S.M.); thomas.cho@westernu.edu (T.C.); timothy.nguyen@westernu.edu (T.N.); gteskey@westernu.edu (G.T.); 3 Department of Basic Medical Sciences, College of Osteopathic Medicine of the Pacific, Western University of Health Sciences, Pomona, CA 91766-1854, USA; rcao@westernu.edu

**Keywords:** *M. tb*, BCG vaccination, immune exhaustion, glutathione, cytokines, granulomas

## Abstract

*Mycobacterium tuberculosis* (*M. tb*) is the etiological agent that is responsible for causing tuberculosis (TB). Although every year *M. tb* infection affects millions of people worldwide, the only vaccine that is currently available is the Bacille Calmette–Guérin (BCG) vaccine. However, the BCG vaccine has varying efficacy. Additionally, the first line antibiotics administered to patients with active TB often cause severe complications and side effects. To improve upon the host response mechanism in containing *M. tb* infection, our lab has previously shown that the addition of the biological antioxidant glutathione (GSH) has profound antimycobacterial effects. The aim of this study is to understand the additive effects of BCG vaccination and *ex-vivo* GSH enhancement in improving the immune responses against *M. tb* in both groups; specifically, their ability to mount an effective immune response against *M. tb* infection, maintain CD4^+^ and CD8^+^ T cells in the granulomas, their response to liposomal glutathione (L-GSH), with varying suboptimal levels of the first line antibiotics isoniazid (INH) and pyrazinamide (PZA), the expressions of programmed death receptor 1 (PD-1), and their ability to induce autophagy. Our results revealed that BCG vaccination, along with GSH enhancement, can prevent the loss of CD4^+^ and CD8^+^ T cells in the granulomas and improve the control of *M. tb* infection by decreasing the expressions of PD-1 and increasing autophagy and production of the cytokines interferon gamma IFN-γ and tumor necrosis factor-α (TNF-α).

## 1. Introduction

Tuberculosis (TB), caused by *Mycobacterium tuberculosis (M. tb)*, continues to afflict millions of people worldwide. In 2017, approximately 10 million people suffered from active TB and 1.6 million died from this disease [1]. Additionally, one third of the world’s population is latently infected with *M. tb*. Individuals infected with *M. tb* have a 5–15% lifetime risk of developing an active disease; however, immunocompromised patients, such as people living with diabetes, malnutrition, human immunodeficiency virus (HIV), or those who use tobacco, have a higher risk of developing active TB [1]. Common symptoms of active pulmonary TB are coughs with a bloody sputum, chest pains, weakness, weight loss, fever, and night sweats, eventually resulting in death when untreated [1]. *M. tb* infection occurs due to inhalation of infectious aerosolized droplets, and the bacteria become seeded in the lower respiratory tract where there is an enrichment of alveolar macrophages.

*M. tb* infection is initiated when the inhaled organisms are phagocytosed by these alveolar macrophages [2]. In an immune-competent individual, the immune system is able to mount a formidable response against *M. tb*, resulting in the formation of a solid and robust granuloma. Composed of a compact aggregate of immune cells [3]. Mature macrophages in the granuloma can fuse into multinucleated giant cells or differentiate into foam cells and epithelioid cells [3]. Alongside macrophages, other cells, such as neutrophils, dendritic cells, natural killer cells, fibroblasts, CD4^+^ T cells, and cytotoxic CD8^+^ T cells, are also recruited into the granuloma via cytokine mediation, leading to containment of the *M. tb* infection [3]. The effector responses inside the granulomas along with a lack of nutrients and oxygen causes *M. tb* to become dormant and remain latent in a nonreplicating state. The contained *M. tb* within a granuloma in the lungs is commonly referred to as latent tuberculosis (LTBI). A breakdown of immune responses designed to contain the infection in immunocompromised individuals can result in reactivation of *M. tb* [4]. This dysregulation promotes liquification of caseum and replication of *M. tb,* thereby promoting cavity formation and the release of *M. tb* to the exterior during coughing, ultimately spreading the infection to other parts of the lungs [5]. Active *M. tb* is able to deflect many host defense mechanisms via the cord factor, preventing phagosome-lysosome fusion and the degradation of the bacilli [6].

In order to effectively contain *M. tb* within the granuloma, proper cytokine-mediated signaling is essential to promote the necessary aggregation of cells [7]. Cytokines, such as interferon gamma (IFN-γ) and tumor necrosis factor-α (TNF-α), play a critical role in both the innate and adaptive immune responses against *M. tb* infection [8]. TNF-α produced by macrophages induces the formation and maintenance of the granuloma. The T-helper 1 (Th1) subset of CD4^+^ T cells releases IFN-γ to activate effector mechanisms in macrophages to not only kill *M. tb* intracellularly but also enhance the effector functions of natural killer cells and cytotoxic T lymphocytes (CD8^+^) T-cells [9].

Once activated, CD8^+^ T cells and natural killer cells will then produce antimicrobial peptides, perforin and granulysin, to destroy intracellular *M. tb* and the host cells.

The programmed death receptor 1 (PD-1), a negative regulator of activated T cells, is markedly upregulated on the surface of pathogen-specific CD8^+^ T cells in mice [10]. Blockage of this pathway restores the CD8^+^ T cell function and reduces the microbial load [10]. PD-1 is also expressed on the surface of CD4^+^ T cells, with a positive correlation in regard to the microbial load and an inverse correlation with the CD4^+^ T cell count.

Although the immune system has a robust defense system in place to combat *M. tb* infection, it cannot always contain the infection. For this reason, host directed therapy is often required.

The Center for Disease Control (CDC) recommends four anti-TB agents to form the core of the treatment regimen for patients with active TB [11]. These drugs include isoniazid (INH), rifampicin (RIF), ethambutol (EMB), and pyrazinamide (PZA). This extensive drug regimen often leads to noncompliance to the TB treatment, leading to multidrug-resistant (MDR)-TB, which is typically resistant to both INH and RIF [12].

Although a myriad of antibiotics can be used to treat this mycobacterial infection, there is only one vaccine available to prime the immune system against *M. tb,* and that is the *Mycobacterium bovis* bacille Calmette–Guérin (BCG) vaccine [13]. BCG used in this vaccine is an attenuated strain of *M. bovis* [13]. The WHO recommends that infants in countries with a high risk of *M. tb* infection be immunized with the BCG vaccine soon after birth [13]. Because the incidence of TB is low in the United States, it is not recommended for infants to be administered this vaccination. Additionally, estimates of the protective efficacy of the BCG vaccine against adult pulmonary TB very widely, ranging from 0 to 80% [13].

Glutathione (GSH), a tripeptide antioxidant composed of glutamine, cysteine, and glycine, is found ubiquitously amongst all cell types. GSH prevents cellular damage by detoxifying reactive oxygen species (ROS) [14]. GSH exists in both a reduced state (rGSH) and an oxidized form (GSSG) [14]. rGSH contains the antioxidant properties, while GSSG is a simple byproduct of the oxidation of GSH and has no antioxidant effects [14]. When exposed to ROS, two molecules of rGSH are converted to GSSG and water [14]. Mycobacteria possess an alternative thiol, mycothiol, rather than GSH, to regulate their redox homeostasis [15]. Due to this property, the presence of millimolar concentrations of GSH (physiological concentrations) inside infected macrophages can lead to inhibition in the growth of *M. tb* [15]. Our laboratory has previously demonstrated that GSH-enhancement by N-acetyl cysteine (NAC) supplementation resulted in a significant reduction of *M. tb* burden among both healthy and diabetic individuals [15]. Additionally, enhancement of GSH by means of NAC has the potential implications of not only reducing the toxicity of anti-TB medications through GSH’s redox potential but may possibly permit lower antibiotic dosage to promote enhanced patient compliance [15]. For this reason, our lab tested the effects of liposomal glutathione (L-GSH) in the presence and absence of sub-optimal concentration of INH and PZA in improving the ability of immune cells isolated from BCG-vaccinated and non-vaccinated individuals to control *M. tb* infection.

In this study, we determined the additive effects of BCG vaccination and *in vitro* GSH-enhancement in improving the ability of immune cells to control *M. tb* infection by measuring the differences in the immune responses between vaccinated and non-vaccinated individuals. Fluorescent staining and other antibody assays were also performed to determine the underlying mechanistic differences in the ability of immune cells from vaccinated and unvaccinated groups to respond to L-GSH, cytokine production, the surface expression of CD4, CD8, PD-1, and their ability to induce autophagy.

## 2. Materials and Methods

### 2.1. Peripheral Blood Mononuclear Cell Isolation

Peripheral blood mononuclear cells (PBMCs) were isolated from the whole blood of both BCG-vaccinated and non-BCG-vaccinated participants. Whole blood was layered in a 1:1 ratio onto ficoll histopaque (Sigma, St. Louis, MO, USA), a high density-pH neutral polysaccharide solution, for density gradient centrifugation (1800 rpm for 30 min) [15]. The PBMCs at the interface were aspirated, washed twice with sterile 1X PBS (Sigma, St. Louis, MO, USA), and resuspended in Roswell Park Memorial Institute (RPMI) (Sigma, St Louis, MO, USA) with 5% human AB serum (Sigma, St. Louis, MO, USA). PBMC counts were determined by trypan blue exclusion staining.

### 2.2. Generation of In Vitro Granulomas

Our laboratory had successfully established an *in vitro* human granuloma model using PBMCs, isolated from healthy subjects and individuals with type 2 diabetes [15,16,17,18]. These granulomas [15,16,17,18] exhibit a physically well-demarcated aggregation of mononuclear cells with a denser central core descending towards the periphery, which can be seen in the in vitro granulomas. Multi-nucleated giant cells (MNGs hallmark of granulomas), T cells, and activated macrophages were also seen. These features are reminiscent of early stage, cellular lung granulomas in experimental animal models of TB, including rabbits and non-human primates. Such granulomas are also noted in the lungs of mice during chronic *M. tb* infection. Using our previously published protocol, we developed in vitro granulomas for the current study. Isolated PBMCs from the two study groups resuspended in RPMI were infected with the Erdman strain of *M. tb* at a multiplicity of infection (MOI) of 0.1:1 cell ratio. 500 µL of the cell suspension containing PBMCs and *M. tb* were added to the 24-well plates. To ensure proper adhesion of isolated immune cells, 24-well plates (Corning, Corning, NY, USA) were coated with 0.001% poly-lysine (Sigma, St. Louis, MO, USA) overnight [15,16]. PBMCs (6 × 10^5^ cells/well) were distributed into the poly-lysine coated 24-well plates [15,16]. PBMCs in the wells were either sham-treated or treated with the minimum inhibitory concentration (MIC), a 1.10 dilution, and a 1.100 dilution of two first-line antibiotics with and without 120 µM of liposomal glutathione (L-GSH (Your Energy Systems)). This comprised INH (0.125 micrograms/mL) standalone, 120 µM of L-GSH, 1/10 INH (0.0125 micrograms/mL) standalone, 120 µM of L-GSH, 1/100 INH (0.00125 micrograms/mL) standalone, 120 µM of L-GSH, PZA (50 micrograms/mL), 120 µM of L-GSH, 1/10 PZA (5 micrograms/mL) standalone, or 120 µM of L-GSH and 1/100 PZA (0.5 micrograms/mL) standalone or 120 µM of L-GSH. All tissue culture plates with infected PBMCs were maintained at 37 °C with 5% CO_2_ until they were terminated at 8 days post infection.

### 2.3. Termination of Granulomas

Following 8 days post infection, the minimum time needed for granuloma formation, *in vitro* granulomas were terminated to determine the intracellular survival of *M. tb* [15,16]. To terminate, the supernatants of each category were aspirated and collected into eppendorf tubes separated by a treatment group, and 250 µL of ice cold, sterile 1× PBS was replaced in lieu of the supernatants followed by gentle scraping of the wells [15,16]. Scraping was done to ensure maximum recovery of granuloma lysates from the wells. 

### 2.4. Colony Forming Units

Collected supernatants and lysates from termination were plated on 7H11 agar media (Hi Media, Santa Maria, CA, USA) enriched with Albumin Dextrose Complex (ADC) (GEMINI, Calabasas, CA, US). They were incubated for a minimum of 3 weeks and 3 days to evaluate the mycobacterial survival under the different treatment conditions by counting the colony forming units (CFUs).

### 2.5. Cytokine Measurements

To measure cytokine levels, the sandwich enzyme-linked immunosorbent assay (ELISA) technique was used. The assay was performed via the manufacturer’s protocol (Invitrogen, Carlsbad, CA, USA). The cytokines measured were IFN-γ and TNF-α in the supernatants at 8 days post-infection to determine the effects of the antibiotics with and without L-GSH treatments on cytokine levels in BCG-vaccinated and non-vaccinated individuals.

### 2.6. Glutathione Measurements

Levels of GSH from the granulomas of non-vaccinated and BCG-vaccinated subjects were measured by the colorimetric method using an assay kit from Arbor Assay (K006-H1). Granuloma lysates were mixed in a 1:1 ratio with cold 5% sulfosalicylic acid (SSA), incubated for 10 min at 4 °C, followed by centrifugation at 14,000 rpm for 10 min. The GSH was measured in the lysates following the manufacturer’s instructions. The reduced GSH (rGSH) was calculated by subtracting the oxidized glutathione (GSSG) from the total GSH. 

### 2.7. Staining and Imaging Techniques

Each trial contained designated wells for fluorescent and light microscopic studies. Cover glasses were allotted into 24-well plates for granuloma formation observation. The cover glasses were fixed with 4% paraformaldehyde (PFA) for 1 h at room temperature and washed three times with 1× PBS for 5 min to remove cell debris. Fixed granulomas were then stained with Hematoxylin and Eosin (H&E) (Poly Scientific, Bay Shore, NY, USA) for 2 min at room temperature and destained with deionized water. The granuloma-stained cover glasses were mounted onto glass slides with HistoChoice mounting media. Fixed granulomas on cover glasses were also permeabilized with Triton X for 2 min and stained overnight with antibodies conjugated with fluorescent markers (CD4-PE, CD8-PE, and LC3B-PE). Cover glasses were washed with phosphate buffer saline (PBS) and mounted on clean glass slides with mounting media containing 4’,6-diamidino-2-phenylindole DAPI For PD1 staining, fixed granulomas on cover glasses were permeabilized with Triton X for 2 min and incubated overnight with anti-PD1 (Pro-Sci), followed by incubation for another 2 h with c-Myc. Cover glasses were then incubated overnight with secondary antibodies (mouse anti-human) and conjugated with fluorescein isothiocyanate (FITC). Cover glasses were mounted using a mounting media containing DAPI. Slides were observed under the fluorescent microscope. Fluorescent images were captured, and the fluorescent intensity was quantified using the ImageJ software (version 8, GraphPad, San Diego, CA, USA).

### 2.8. Statistical Analysis

Statistical data analysis was performed using GraphPad Prism Software 8 using the unpaired t-test with Welch correction for two sampled graphs. A one-way ANOVA (analysis of variance) was performed for samples with greater than two categories with Tukey corrections. Reported values are the means with each respective category. A *p* < 0.05 was considered significant. The *p* value style consisted of 0.1234 as not significant, 0.0332 with one asterisk (*), 0.0021 with two asterisks (**), 0.0002 with three asterisks (***), and less than 0.0001 with four asterisks (****). A hash mark (#) indicates categories compared to control, and an asterisk indicates categories compared to the previous category directly before it.

## 3. Results

### 3.1. Survival of the Erdman Strain of M. tb in the In Vitro Granulomas

**Figure 1:**
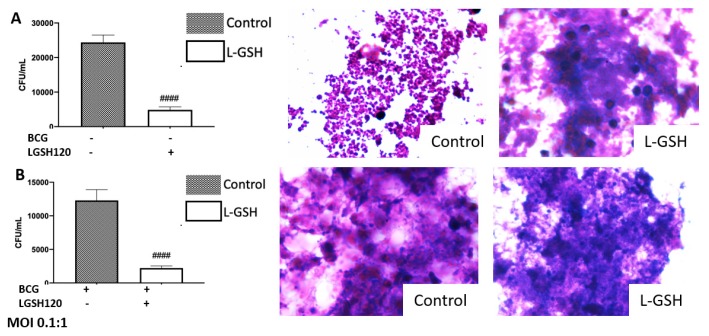

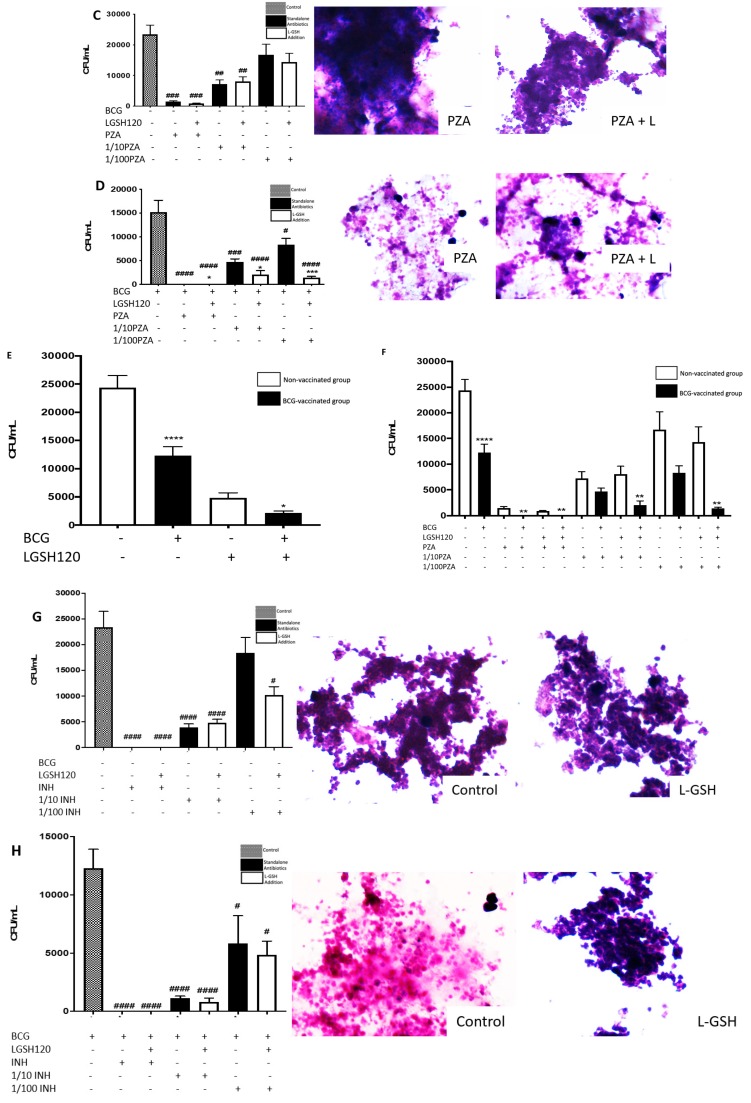

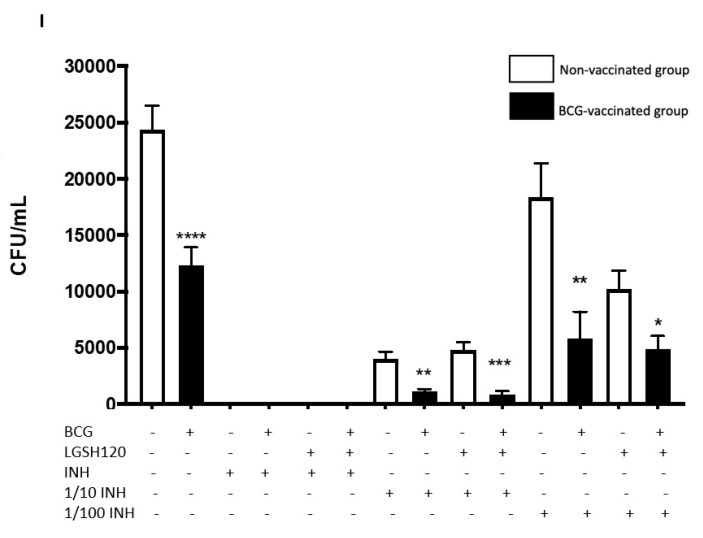
Survival of the Erdman strain of *Mycobacterium tuberculosis* treated with Pyrazinamide and Isoniazid in media. (**A**) *M. tb* growth in 7H11 agar media from non-vaccinated subjects with liposomal glutathione (L-GSH) treatment along with Hematoxylin and Eosin (H&E) images of granuloma like structures from eight-day terminated samples; (**B**) *M. tb* growth in 7H11 agar media from Bacille Calmette–Guérin (BCG) vaccinated subjects with L-GSH treatment along with H&E images of granuloma-like structures from eight-day terminated samples; (**C**) *M. tb* growth in 7H11 agar media with pyrazinamide (PZA), 1/10 PZA, and 1/100 PZA treatment in the presence or absence of L-GSH in non-vaccinated subjects; (**D**) *M. tb* growth in 7H11 agar media with PZA, 1/10 PZA, and 1/100 PZA treatment in the presence or absence of L-GSH in BCG-vaccinated subjects. (**E**) *M. tb* growth in 7H11 agar media from non-vaccinated (white bar) and BCG-vaccinated (black bar) groups with sham treatment and L-GSH treatment; (**F**) *M. tb* growth in 7H11 agar media in non-vaccinated and BCG-vaccinated groups with PZA treatment. Data represents ± SE(standard error) rom experiments performed from 14 different subjects. An unpaired t-test with Welch corrections was used in Figure (**A**). Analysis of Figures B–F utilized a one-way ANOVA (analysis of variance) with Tukey test. The *p* value style consisted of 0.1234 as not significant, 0.0332 with one asterisk (*), 0.0021 with two asterisks (**), 0.0002 with three asterisks (***), and less than 0.0001 with four asterisks (****). A hash mark (#) indicates categories compared to control, and an asterisk indicates categories compared to the previous category directly before it. (**G**) *M. tb* growth in 7H11 agar media with isoniazid (INH), 1/10 INH, and 1/100 INH treatment in the presence or absence of L-GSH in non-vaccinated subject; (**H**) *M. tb* growth in 7H11 agar media with INH, 1/10 INH, and 1/100 INH treatment in the presence or absence of L-GSH in BCG-vaccinated subjects; (**I)** *M. tb* growth in 7H11 agar media in non-vaccinated and BCG-vaccinated groups with INH treatment. Data represents ± SE from experiments performed from 14 different subjects. Analysis of figures utilized a one-way ANOVA with Tukey test. The *p* value style consisted of 0.1234 as not significant, 0.0332 with one asterisk (*), 0.0021 with two asterisks (**), 0.0002 with three asterisks (***), and less than 0.0001 with four asterisks (****). A hash mark (#) indicates categories compared to control, and an asterisk indicates categories compared to the previous category directly before it.

We first tested the effects of L-GSH in controlling the growth of *M. tb* inside in vitro granulomas derived from non-vaccinated and BCG-vaccinated subjects. Approximately, 25 µL of granuloma lysates were plated on 7H11 growth media and incubated for four weeks to allow adequate time for *M. tb* growth. In both non-vaccinated and BCG-vaccinated individuals, there was a significant reduction in the bacterial load when standalone L-GSH was added (Figure 1A,B). We then measured the effects of PZA added at various concentrations in the presence and absence of L-GSH in altering the viability of *M. tb*. In the non-vaccinated group, there was a significant reduction in the viability of *M. tb* when PZA was added at MIC and at the 1/10 lower dilution in the presence and absence of L-GSH when compared to the untreated control (Figure 1C). In the BCG-vaccinated group, there was a significant reduction in the viability of *M. tb* with the addition of L-GSH at all tested concentrations of PZA when compared to the untreated control category and to the PZA-alone treated groups (MIC, 10 and 100 times lower than MIC concentrations). Notably, in the BCG-vaccinated group, PZA + L-GSH 120 resulted in complete clearance of *M. tb* (Figure 1D). Hematoxylin and Eosin staining was performed to observe the morphology of the granuloma-like structures. Not surprisingly, we witnessed more solid and robust granulomas when the bacterial load was higher. Correspondingly, the aggregation of the granuloma was not as dense when the infection was cleared (Figure 1A,B). Additionally, we compiled the results from the *M. tb* survival assays from the non-vaccinated and BCG-vaccinated individuals to compare the same treatment categories. The BCG-vaccinated subjects displayed a significantly greater ability to kill *M. tb* and/or containment potential than the non-vaccinated control. L-GSH treatment resulted in a similar trend, exhibiting improved killing of *M. tb* in the granulomas from BCG-vaccinated individuals when compared to the non-vaccinated subjects (Figure 1E). In regard to the comparison of the PZA treated granulomas from non-vaccinated and BCG-vaccinated subjects, there was a significant reduction in the viability of *M. tb* in all categories of the BCG-vaccinated individuals when compared to the non-vaccinated individuals of the same treatment category (Figure 1F).

The effects of INH in altering the viability of *M. tb* in the granulomas of non-vaccinated and BCG-vaccinated subjects was also tested. In the non-vaccinated group, there was a significant reduction in the viability of *M. tb* (almost undetectable colonies) when INH was added at MIC. There was a significant reduction in CFUs in all categories, excluding the 1/100 INH category (Figure 1G). In the BCG-vaccinated group, there was a significant reduction in the viability of *M. tb* at MIC and in all categories (Figure 1H). When comparing INH treated granulomas in non-vaccinated and BCG-vaccinated subjects, granulomas from BCG-vaccinated subjects controlled the *M. tb* infection more effectively compared to the non-vaccinated groups in the presence and absence of INH and/or L-GSH (Figure 1I).

### 3.2. Levels of the Reduced Form of GSH in the In Vitro Treated Granulomas

**Figure 2:**
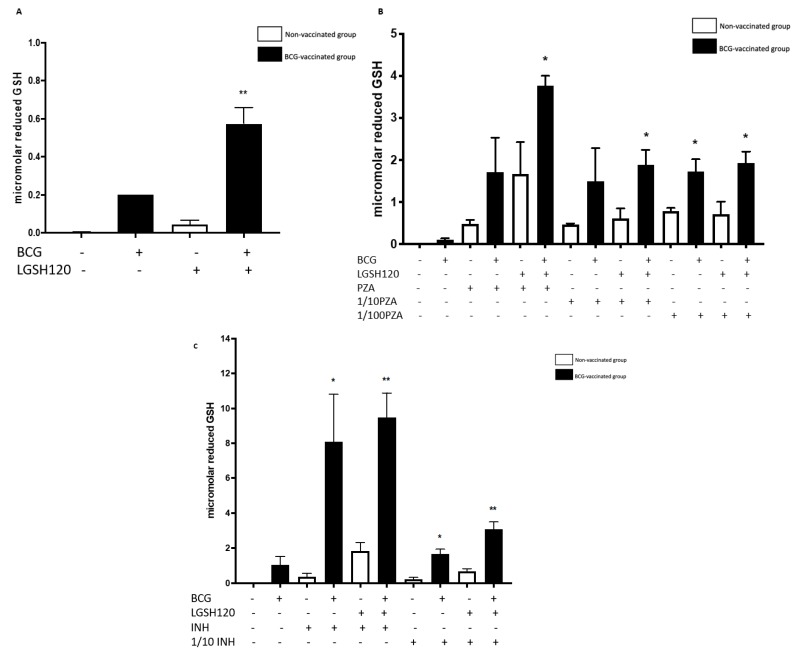
Levels of reduced GSH in PZA and INH treated granulomas. The GSH assay was performed by the colorimetric method using an Arbor Assays kit. (**A**) Comparison of reduced GSH levels in non-vaccinated and BCG-vaccinated groups; (**B**) GSH measurements in PZA treated granulomas from non-vaccinated and BCG-vaccinated subjects. Data represents ±SE from experiments performed from 14 different subjects. The *p* value style consisted of 0.1234 as not significant, 0.0332 with one asterisk (*), 0.0021 with two asterisks (**), 0.0002 with three asterisks (***), and less than 0.0001 with four asterisks (****). (**C**) The GSH assay was performed by the colorimetric method using an Arbor Assays kit. GSH measurements in INH treated granulomas from non-vaccinated and BCG-vaccinated subjects. Data represents ± SE from experiments performed from 14 different subjects. Analysis of figures utilized a one-way ANOVA with Tukey test. The *p* value style consisted of 0.1234 as not significant, 0.0332 with one asterisk (*), 0.0021 with two asterisks (**), 0.0002 with three asterisks (***), and less than 0.0001 with four asterisks (****).

GSH levels were measured from the cellular lysates of the PBMCs using an assay kit from Arbor Assays. The untreated granulomas from BCG-vaccinated individuals displayed higher levels of the reduced form of GSH than non-vaccinated individuals. L-GSH treatment resulted in increased levels of GSH in both the groups; however, there was a significant increase in the levels of GSH from the granulomas of BCG-vaccinated individuals (Figure 2A). Treatment of granulomas from BCG-vaccinated subjects with PZA in combination with L-GSH resulted in a significant increase in the levels of GSH compared to the non-vaccinated group (Figure 2B). The levels of reduced forms of GSH were also measured in the lysates of the eight-day terminated samples. In granulomas treated with INH, BCG-vaccinated individuals had a significant increase in GSH in all categories in the presence and absence of L-GSH (Figure 2C).

### 3.3. Expression of CD4 in the In Vitro Granulomas

**Figure 3:**
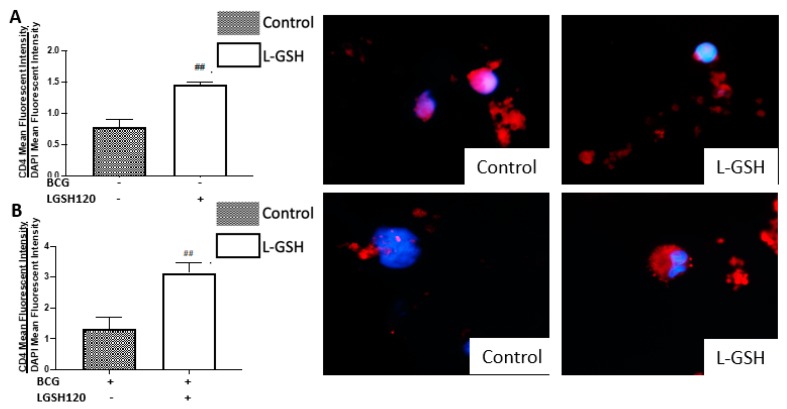

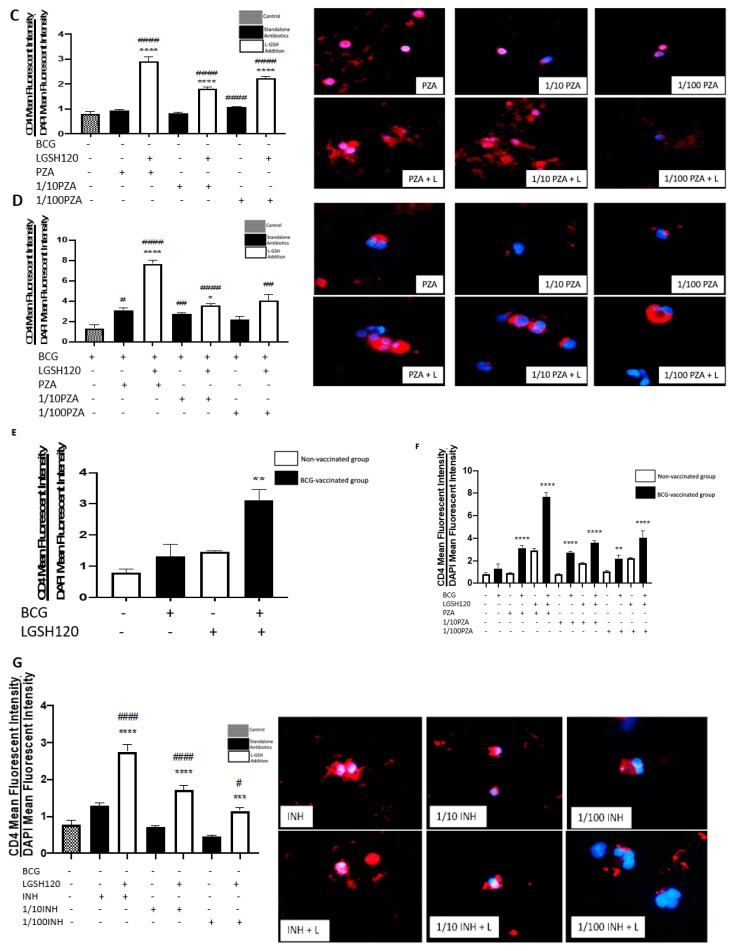

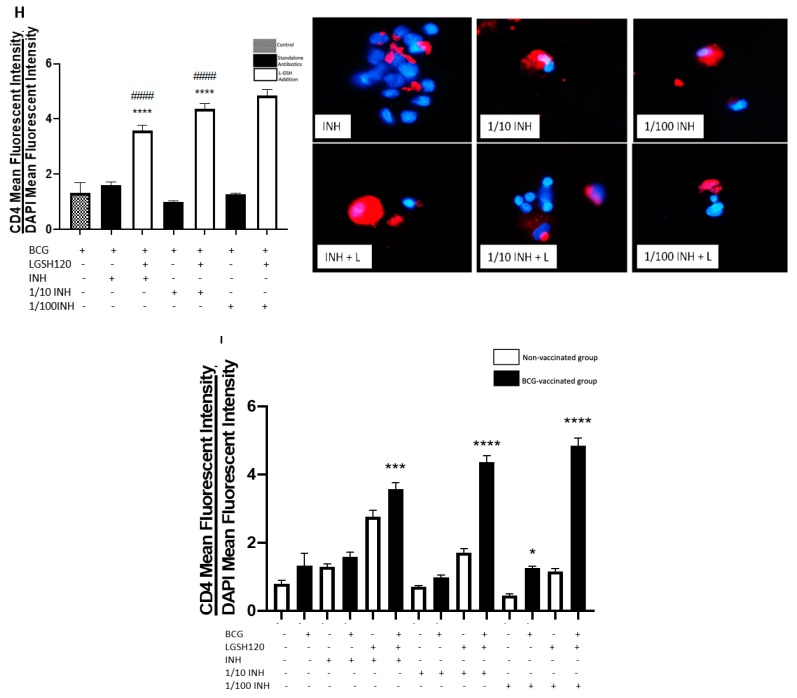
Expression of CD4 in PZA and INH treated granulomas. CD4 T cells were measured with an anti-CD4 antibody conjugated with (Phycoerythrin-Cy5) PE-Cy5. The nuclei of cells were stained with (4’,6-diamidino-2-phenylindole) DAPI. The mean fluorescence of CD4 was corrected with the mean DAPI fluorescence. (**A**) CD4 mean fluorescence from non-vaccinated subjects with L-GSH treatment along with CD4 fluorescent images; (**B**) CD4 mean fluorescence from BCG vaccinated subjects with L-GSH treatment along with CD4 fluorescent images; (**C**) CD4 mean fluorescence with PZA, 1/10 PZA, and 1/100 PZA treatment in the presence or absence of L-GSH in non-vaccinated subjects; (**D**) CD4 mean fluorescence with PZA, 1/10 PZA, and 1/100 PZA treatment in the presence or absence of L-GSH in BCG-vaccinated subjects; (**E**) CD4 mean fluorescence in non-vaccinated and BCG-vaccinated groups with sham treatment and L-GSH treatment; (**F**) CD4 mean fluorescence in non-vaccinated and BCG-vaccinated groups with PZA treatment. Data represents ± SE from experiments performed from 14 different subjects. An unpaired t-test with Welch corrections was used in Figure (**A**). Analysis of Figures B–F utilized a one-way ANOVA with Tukey test. The *p* value style consisted of 0.1234 as not significant, 0.0332 with one asterisk (*), 0.0021 with two asterisks (**), 0.0002 with three asterisks (***), and less than 0.0001 with four asterisks (****). A hash mark (#) indicates categories compared to control, and an asterisk indicates categories compared to the previous category directly before it. (**G**) CD4 mean fluorescence with INH, 1/10 INH, and 1/100 INH treatment in the presence or absence of L-GSH in non-vaccinated subjects; (**H**) CD4 mean fluorescence with INH, 1/10 INH, and 1/100 INH treatment in the presence or absence of L-GSH in BCG-vaccinated subjects; (**I**) CD4 mean fluorescence in non-vaccinated and BCG-vaccinated groups with INH treatment. Data represents ± SE from experiments performed from 14 different subjects. The analysis of figures utilized a one-way ANOVA with Tukey test. The *p* value style consisted of 0.1234 as not significant, 0.0332 with one asterisk (*), 0.0021 with two asterisks (**), 0.0002 with three asterisks (***), and less than 0.0001 with four asterisks (****). A hash mark (#) indicates categories compared to the control, and an asterisk indicates categories compared to the previous category directly before it.

CD4 T cell expression was measured with an anti-CD4 antibody, conjugated with Phycoerythrin-Cy5 (PE-Cy5). Analysis of CD4 T cells were corrected with the average mean fluorescence of 4’,6-diamidino-2-phenylindole (DAPI). The addition of standalone L-GSH resulted in a significant increase of the CD4 mean fluorescence in both vaccinated and non-vaccinated groups (Figure 3A,B). CD4 expression levels were then measured from the pyrazinamide treatment categories in the presence and absence of L-GSH, both in the non-vaccinated group and the BCG-vaccinated group. In the non-vaccinated group, there was a significant increase in CD4 expression with L-GSH addition when compared to both the control group and the standalone PZA. A dilution of 1/100 PZA standalone treatment resulted in a significant increase in CD4 expression when compared to the control (Figure 3C). In the BCG-vaccinated group, L-GSH addition resulted in a significant increase in CD4 expression when compared to the sham treated group. The categories of standalone MIC PZA and 1/10 PZA resulted in a significant increase in CD4 expression when compared to the control. Additionally, there was a significant increase in the expression of CD4 in PZA + L-GSH and 1/10PZA + L-GSH categories when compared to their standalone counterparts (Figure 3D). The compiled graph comparing the sham treated versus L-GSH treated granulomas from non-vaccinated and BCG-vaccinated subjects demonstrated a significant increase in the mean fluorescent intensity of CD4 in BCG-vaccinated subjects with the addition of L-GSH (Figure 3E). In comparison to the non-vaccinated group, PZA treatment resulted in a significant increase in CD4 expression in the granulomas from BCG-vaccinated groups (Figure 3F). L-GSH + PZA treatment resulted in a further increase in the expression of CD4 in the granulomas from BCG-vaccinated subjects compared to the non-vaccinated group (Figure 3F).

There is an increase in the viability potential of CD4 when L-GSH adjunctive treatment is added. In the compiled data comparing the non-vaccinated group to the BCG-vaccinated group, in INH treated granulomas, there was a significant increase in CD4 expression in the BCG-vaccinated groups in most categories, except for MIC and 1/10 INH + L-GSH (Figure 3G,H).

### 3.4. Expression of CD8 in the In Vitro Granulomas

**Figure 4:**
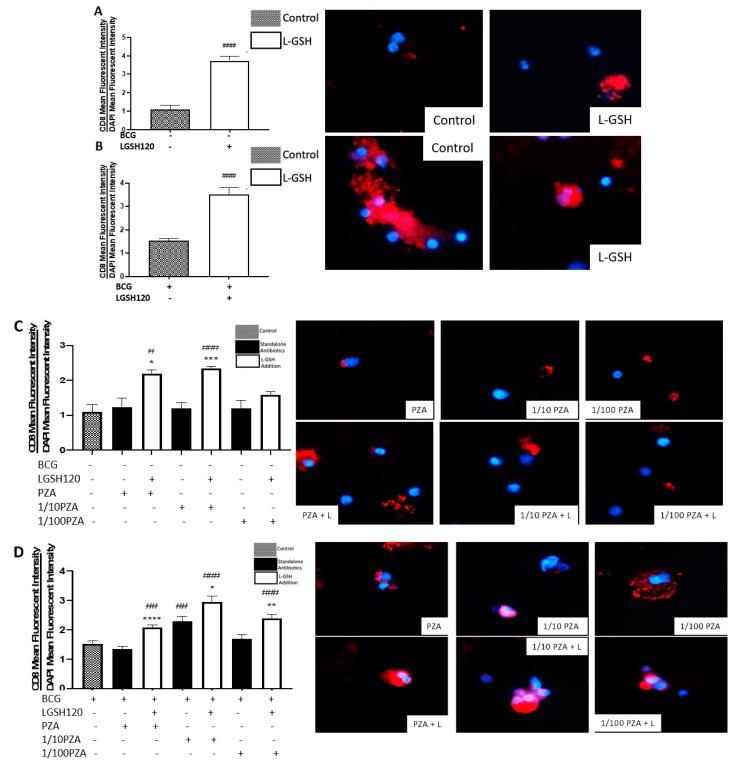

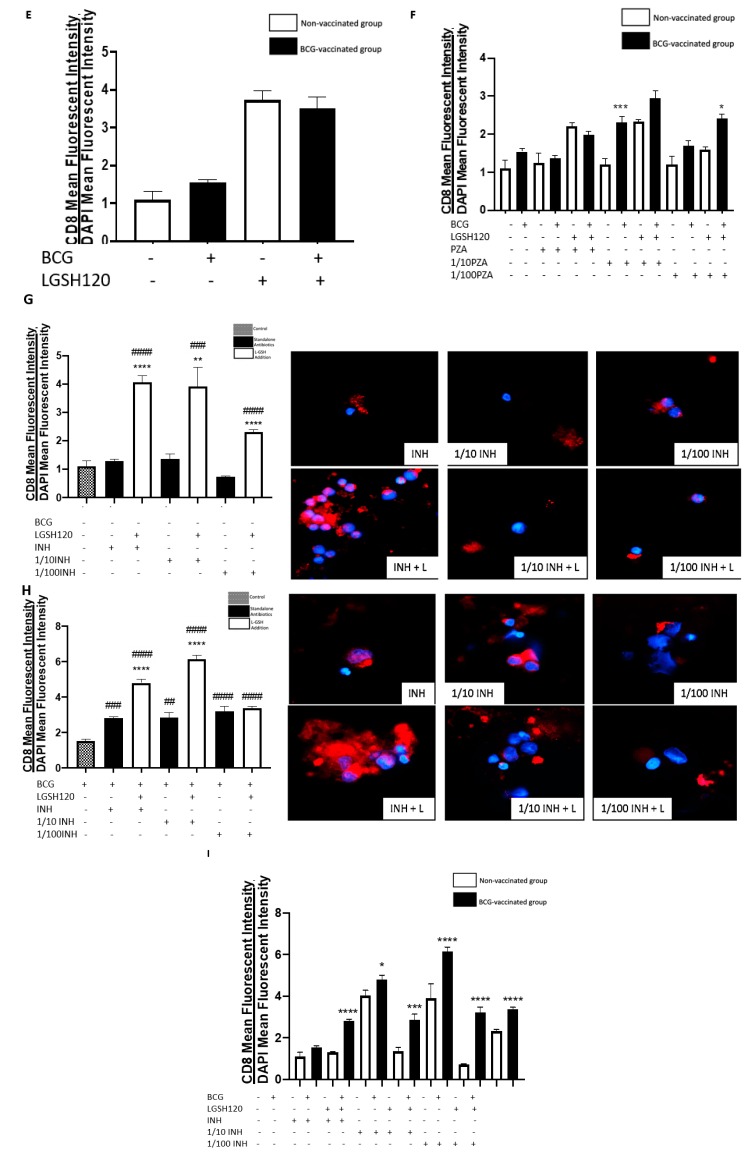
Expression of CD8 in PZA and INH treated granulomas. CD8 T cells were measured with an anti-CD8 antibody conjugated with PE-Cy5. The nuclei of cells were stained with DAPI. The mean fluorescence of CD8 was corrected with the mean DAPI fluorescence. (**A**) CD8 mean fluorescence from non-vaccinated subjects with L-GSH treatment along with CD4 fluorescent images; (**B**) CD8 mean fluorescence from BCG-vaccinated subjects with L-GSH treatment along with CD8 fluorescent images; (**C**) CD8 mean fluorescence with PZA, 1/10 PZA, and 1/100 PZA treatment in the presence or absence of L-GSH in non-vaccinated subjects; (**D**) CD8 mean fluorescence with PZA, 1/10 PZA, and 1/100 PZA treatment in the presence or absence of L-GSH in BCG-vaccinated subjects; (**E**) CD8 mean fluorescence in non-vaccinated and BCG-vaccinated groups with sham treatment and L-GSH treatment; (**F**) CD8 mean fluorescence in non-vaccinated and BCG-vaccinated groups with PZA treatment. Data represents ± SE from experiments performed from 14 different subjects. An unpaired t-test with Welch corrections was used in Figure (A). Analysis of Figures B–F utilized a one-way ANOVA with Tukey test. The *p* value style consisted of 0.1234 as not significant, 0.0332 with one asterisk (*), 0.0021 with two asterisks (**), 0.0002 with three asterisks (***), and less than 0.0001 with four asterisks (****). A hash mark (#) indicates categories compared to the control, and an asterisk indicates categories compared to the previous category directly before it. (**G**) CD8 mean fluorescence with INH, 1/10 INH, and 1/100 INH treatment in the presence or absence of L-GSH in non-vaccinated subjects; (**H**) CD8 mean fluorescence with INH, 1/10 INH, and 1/100 INH treatment in the presence or absence of L-GSH in BCG-vaccinated subjects; (**I**) CD8 mean fluorescence in non-vaccinated and BCG-vaccinated groups with INH treatment. Data represents ± SE from experiments performed from 14 different subjects. Analysis of figures utilized a one-way ANOVA with Tukey test. The *p* value style consisted of 0.1234 as not significant, 0.0332 with one asterisk (*), 0.0021 with two asterisks (**), 0.0002 with three asterisks (***), and less than 0.0001 with four asterisks (****). A hash mark (#) indicates categories compared to the control, and an asterisk indicates categories compared to the previous category directly before it.

CD8 T cell expression was measured with an anti-CD8 antibody, conjugated with PE-Cy5. Analysis of CD8 expression was corrected with the average mean fluorescence of DAPI. Individuals vaccinated with BCG displayed the ability to maintain an elevated number of CD8 T cells in the granulomas compared to the non-vaccinated individuals. The addition of L-GSH significantly increased the already high levels of CD8 expression in the granulomas from BCG-vaccinated subjects (Figure 4A,B). A similar trend was found with the PZA treatment groups. Among the non-vaccinated group, the addition of L-GSH + PZA also significantly increased the levels of CD8 expression when compared to the control group and to its counterpart with standalone antibiotics, with the exception of 1/100 PZA + L-GSH (Figure 4C). However, in the BCG-vaccinated group, there was a significant increase in CD8 expression in all the PZA + L-GSH treatment groups (Figure 4D). This can be explained due to the restorative effects L-GSH exhibits to diminish T cell exhaustion. When directly comparing the non-vaccinated to BCG-vaccinated groups, there was no significance between the two sham-treated and L-GSH-treated categories (Figure 4E). The general trend with PZA treatment demonstrates that, on average, BCG-vaccinated subjects possess higher levels of CD8 expression, with significance found in the 1/10 PZA and 1/100 PZA + L-GSH treatment groups (Figure 4F).

In the presence of L-GSH, there was a significant increase in the levels of CD8 T cells compared to the sham-treated and INH alone treated groups in the non-vaccinated group (Figure 4G). In the BCG-vaccinated group, there was a significant increase in CD8 expression in all categories when compared to the control group. Additionally, in the INH + L-GSH and 1/10 INH + L-GSH treatment groups, there was a significant increase when compared to their standalone antibiotic counterparts (Figure 4H). The mean fluorescence of CD8 in INH-treated granulomas showed a significant increase in BCG-vaccinated subjects compared to non-vaccinated subjects in the presence and absence of INH and/or L-GSH treatment (Figure 4I).

### 3.5. Expression of PD-1 in the In Vitro Granulomas

**Figure 5:**
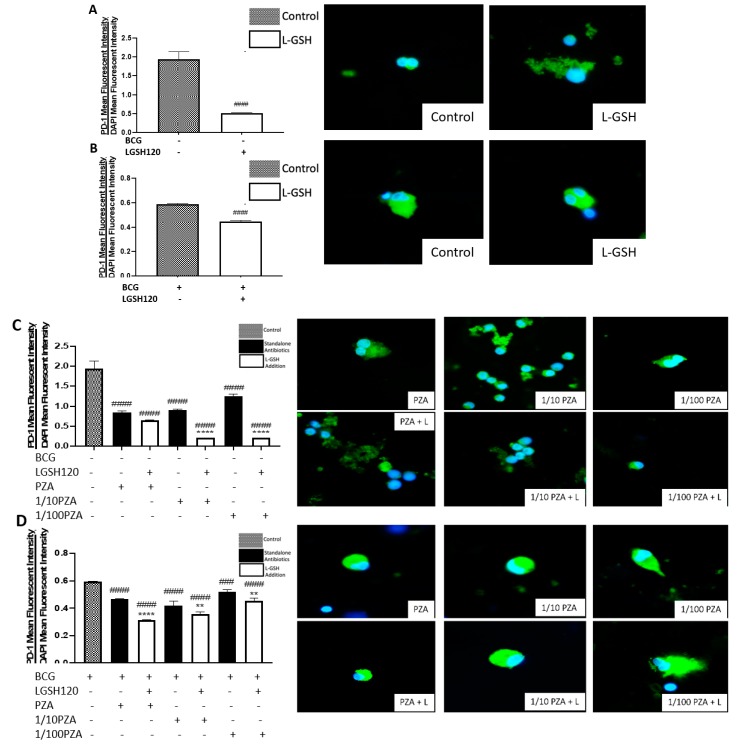

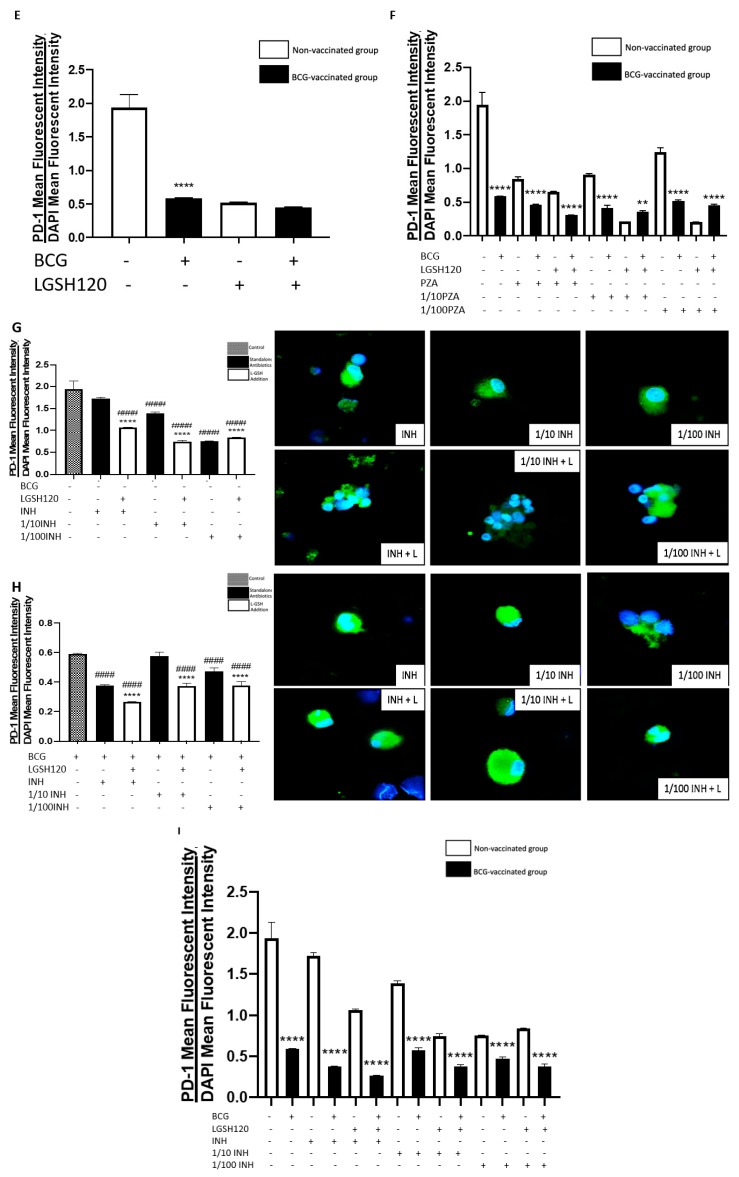
Expression of programmed death receptor 1 (PD-1) in PZA treated granulomas. PD-1 T cells were measured with an anti-PD1 primary monoclonal antibody conjugated with HRP. A secondary anti-mouse PE-Cy5 antibody was added after blocking with 0.1% Triton X for 1 h. The nuclei of cells were stained with DAPI. The mean fluorescence of PD-1 was corrected with the mean DAPI fluorescence. (**A**) PD-1 mean fluorescence from non-vaccinated subjects with L-GSH treatment along with PD-1 fluorescent images; (**B**) PD-1 mean fluorescence from BCG vaccinated subjects with L-GSH treatment along with PD-1 fluorescent images; (**C**) PD-1 mean fluorescence with PZA, 1/10 PZA, and 1/100 PZA treatment in the presence or absence of L-GSH in non-vaccinated subjects; (**D**) PD-1 mean fluorescence with PZA, 1/10 PZA, and 1/100 PZA treatment in the presence or absence of L-GSH in BCG-vaccinated subjects; (**E**) PD-1 mean fluorescence in non-vaccinated and BCG-vaccinated groups with sham treatment and L-GSH treatment; (**F**) PD-1 mean fluorescence in non-vaccinated and BCG-vaccinated groups with PZA treatment. Data represents ± SE from experiments performed from 14 different subjects. An unpaired t-test with Welch corrections was used in Figure (**A**). Analysis of Figures B–F utilized a one-way ANOVA with Tukey test. The *p* value style consisted of 0.1234 as not significant, 0.0332 with one asterisk (*), 0.0021 with two asterisks (**), 0.0002 with three asterisks (***), and less than 0.0001 with four asterisks (****). A hash mark (#) indicates categories compared to the control, and an asterisk indicates categories compared to the previous category directly before it. (**G**) PD-1 mean fluorescence with INH, 1/10 INH, and 1/100 INH treatment in the presence or absence of L-GSH in non-vaccinated subjects; (**H**) PD-1 mean fluorescence with INH, 1/10 INH, and 1/100 INH treatment in the presence or absence of L-GSH in BCG-vaccinated subjects; (**I**) PD-1 mean fluorescence in non-vaccinated and BCG-vaccinated groups with INH treatment. Data represents ± SE from experiments performed from 14 different subjects. Analysis of figures utilized a one-way ANOVA with Tukey test. The *p* value style consisted of 0.1234 as not significant, 0.0332 with one asterisk (*), 0.0021 with two asterisks (**), 0.0002 with three asterisks (***), and less than 0.0001 with four asterisks (****). A hash mark (#) indicates categories compared to the control, and an asterisk indicates categories compared to the previous category directly before it.

Programmed death 1 markers were measured with a conjugated horseradish peroxidase (HRP) anti-PD1 antibody with an anti-mouse FITC secondary antibody. Analysis of PD-1 expression was corrected with the average mean fluorescence of DAPI. Our lab observed a significant decrease in the expression of PD-1 in response to L-GSH treatment in both non-vaccinated and BCG-vaccinated subjects when compared to the sham-treated category (Figure 5A,B). When the granulomas were treated with PZA, there was a significant decrease in PD-1 expression in the non-vaccinated group among all categories when compared to the control group. Additionally, in response to L-GSH, there was a significant decrease in PD-1 expression to the 1/10 PZA + LGSH and 1/100 PZA + L-GSH when compared to their counterparts without L-GSH treatment (Figure 5C). In the BCG-vaccinated group, there was a decrease in PD-1 expression across all categories when compared to the control, and the addition of L-GSH further significantly reduced PD-1 expression when compared to the antibiotics without L-GSH (Figure 5D). Between the non-vaccinated and BCG-vaccinated sham-treated and L-GSH-treated groups, the BCG-vaccinated group had significantly less PD-1 expression in the sham-treated control (Figure 5E). When treated with PZA, the granulomas from BCG-vaccinated subjects demonstrated a significant decrease in PD-1 expression when compared to the non-vaccinated subjects, with the exception of 1/10 PZA + L-GSH and 1/100 PZA + L-GSH (Figure 5F). We observed an overall decreased expression of PD-1 from the granulomas of BCG-vaccinated subjects compared to the non-vaccinated group. The levels of PD-1 were measured in granulomas treated with INH in the presence and absence of L-GSH. In the non-vaccinated group, the addition of L-GSH leads to a significant decrease in PD-1 expression when compared to standalone antibiotics and the control. Additionally, there was a significant decrease of PD-1 expression in the 1/10 INH and 1/100 INH categories when compared to the control (Figure 5G). Similar to the non-vaccinated group, in the BCG-vaccinated group, L-GSH addition to the INH treatment groups leads to a significant decrease in PD-1 expression compared to the sham-treated and standalone antibiotic-treated categories. Additionally, there was a significant decrease of PD-1 in the MIC INH and 1/100 INH categories (Figure 5H). Importantly, the BCG-vaccinated group had significantly lower PD-1 levels at every category (INH treatment group in the presence or absence of L-GSH) when compared to the non-vaccinated group (Figure 5I).

### 3.6. Cytokine Responses in the In Vitro Granulomas

**Figure 6:**
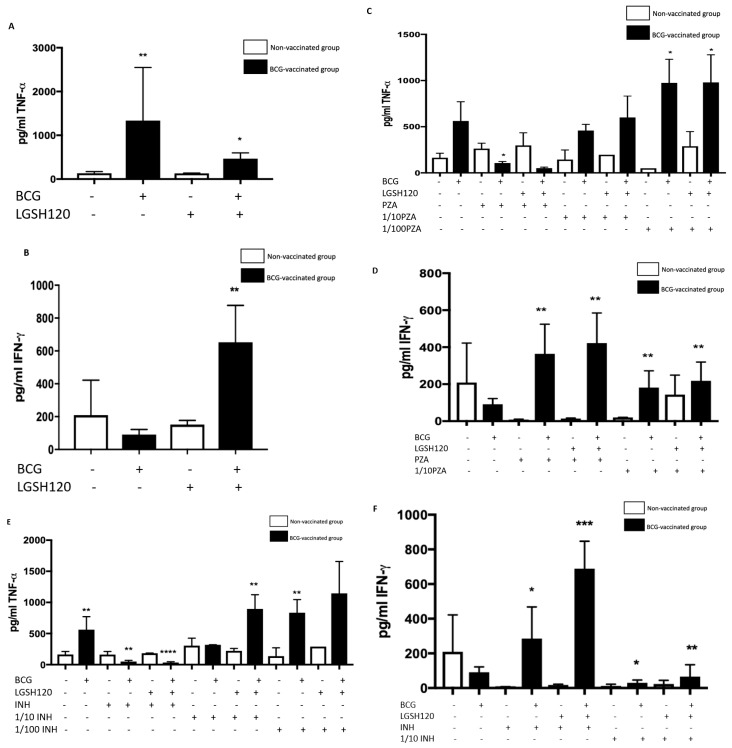
Cytokine responses in PZA and INH treated granulomas. (**A**) Tumor necrosis factor-α (TNF-α) levels from the supernatants of non-vaccinated and BCG-vaccinated sham-treated and L-GSH-treated granulomas at eight days post-infection; (**B**) TNF-α levels from the supernatants of non-vaccinated and BCG-vaccinated PZA treated granulomas at eight days post-infection; (**C**) interferon gamma (IFN-γ) levels from the supernatants of non-vaccinated and BCG-vaccinated sham-treated and L-GSH-treated granulomas at eight days post-infection; (**D**) IFN-γ levels from the supernatants of non-vaccinated and BCG-vaccinated PZA treated granulomas at eight days post-infection. Data represents ± SE from experiments performed from 14 different subjects. Analysis of figures utilized a one-way ANOVA with Tukey test. The *p* value style consisted of 0.1234 as not significant, 0.0332 with one asterisk (*), 0.0021 with two asterisks (**), 0.0002 with three asterisks (***), and less than 0.0001 with four asterisks (****). (**E**) TNF-α levels from the supernatants of non-vaccinated and BCG-vaccinated INH-treated granulomas at eight days post-infection; (**F**) IFN-γ levels from the supernatants of non-vaccinated and BCG-vaccinated INH-treated granulomas at eight days post-infection. Data represents ± SE from experiments performed from 14 different subjects. Analysis of figures utilized a one-way ANOVA with Tukey test. The *p* value style consisted of 0.1234 as not significant, 0.0332 with one asterisk (*), 0.0021 with two asterisks (**), 0.0002 with three asterisks (***), and less than 0.0001 with four asterisks (****).

Using the sandwich Enzyme-linked Immune Sorbent Assay (ELISA) method, we measured the levels of TNF-α, a cytokine that causes the recruitment of other cells to form a granuloma, and IFN-γ, which enhances the effector functions of macrophages to control intracellular *M. tb* infection. These cytokines were measured from the supernatants of the terminated samples. TNF-α expression was significantly increased in the control and L-GSH treatment categories of the BCG-vaccinated group (Figure 6A). When the granulomas were treated with PZA, there was a general upwards trend in TNF-α production from the BCG vaccinated categories, especially in the lower concentrations (Figure 6B). However, due to complete bacterial clearance of the MIC treated categories, no further immunological responses were needed, which is why we ascertain that the TNF-α levels at the lower concentrations were low. There was a significant increase in IFN-γ in the BCG-vaccinated L-GSH treatment group (Figure 6C). PZA treatment in the presence and absence of L-GSH resulted in a significant increase of IFN-γ in the BCG-vaccinated group when compared to the non-vaccinated group (Figure 6D). Increased production of IFN-γ may be the mechanism by which BCG-vaccinated subjects display enhanced control over the bacteria. TNF-α and IFN-γ levels were measured in the supernatants of INH-treated granulomas. TNF-α production was significantly elevated in the control category and in the lower 1/10 INH + L-GSH and 1/100 INH + L-GSH120 categories in BCG vaccinated groups. Treatment with INH at MIC in the presence and absence of L-GSH resulted in complete inhibition in the growth of *M. tb*, leading to homeostasis in the immune response, therefore resulting in significantly lower TNF-α production in BCG-vaccinated subjects (Figure 6E). INH treatment of BCG-vaccinated granulomas in the presence or absence of L-GSH resulted in significantly higher levels of IFN-γ when compared to the non-vaccinated group (Figure 6F). This follows the same trend as those treated with PZA.

### 3.7. Autophagy Levels in the In Vitro Granulomas

**Figure 7:**
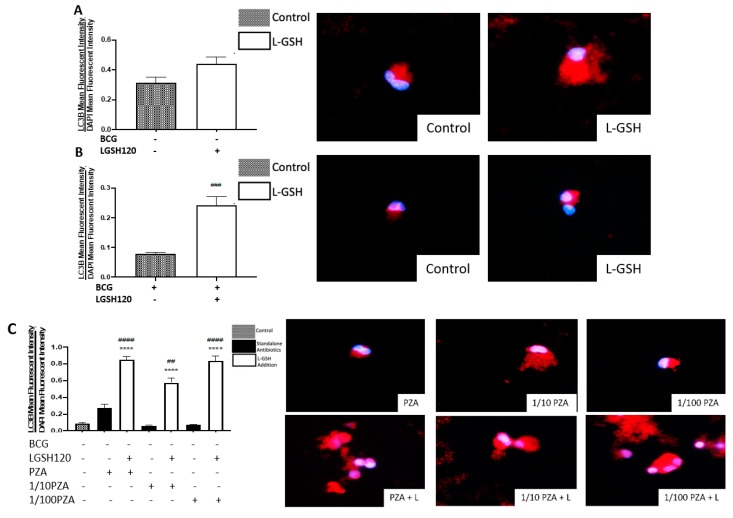

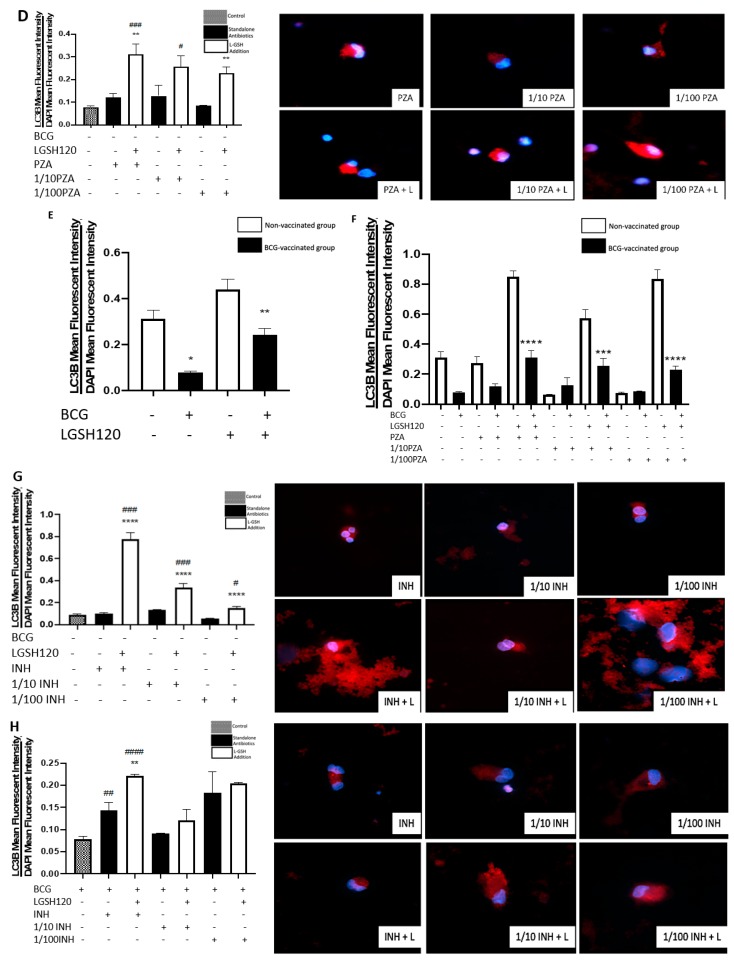

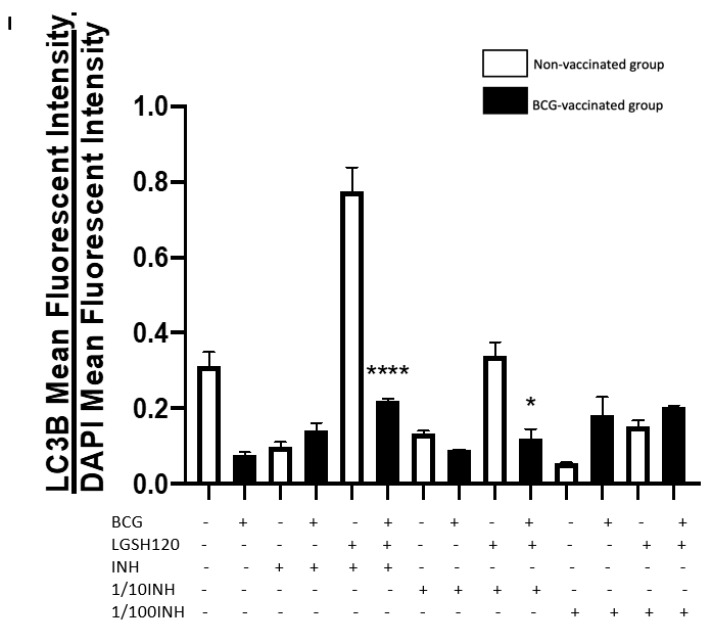
Autophagy levels in PZA- and INH-treated granulomas. Autophagy was measured with an anti-LC3B antibody, conjugated with PE-Cy5. The nuclei of cells were stained with DAPI. The mean fluorescence of LC3B was corrected with the mean DAPI fluorescence. (**A**) LC3B mean fluorescence from non-vaccinated subjects with L-GSH treatment, alongside LC3B fluorescent images; (**B**) LC3B mean fluorescence from BCG-vaccinated subjects with L-GSH treatment, alongside LC3B fluorescent images; (**C**) LC3B mean fluorescence with PZA, 1/10 PZA, and 1/100 PZA treatment in the presence or absence of L-GSH in non-vaccinated subjects; (**D**) LC3B mean fluorescence with PZA, 1/10 PZA, and 1/100 PZA treatment in the presence or absence of L-GSH in BCG-vaccinated subjects; (**E**) LC3B mean fluorescence in non-vaccinated and BCG-vaccinated groups with sham treatment and L-GSH treatment; (**F**) LC3B mean fluorescence in non-vaccinated and BCG-vaccinated groups with PZA treatment. Data represents ± SE from experiments performed from 14 different subjects. An unpaired t-test with Welch corrections was used in Figure (**A**). Analysis of Figures B–F utilized a one-way ANOVA with Tukey test. The *p* value style consisted of 0.1234 as not significant, 0.0332 with one asterisk (*), 0.0021 with two asterisks (**), 0.0002 with three asterisks (***), and less than 0.0001 with four asterisks (****). A hash mark (#) indicates categories compared to the control, and an asterisk indicates categories compared to the previous category directly before it. (**G**) LC3B mean fluorescence with INH, 1/10 INH, and 1/100 INH treatment in the presence or absence of L-GSH in non-vaccinated subjects; (**H**) LC3B mean fluorescence with INH, 1/10 INH, and 1/100 INH treatment in the presence or absence of L-GSH in BCG-vaccinated subjects; (**I**) LC3B mean fluorescence in non-vaccinated and BCG-vaccinated groups with INH treatment. Data represents ± SE from experiments performed from 14 different subjects. Analysis of figures utilized a one-way ANOVA with Tukey test. The *p* value style consisted of 0.1234 as not significant, 0.0332 with one asterisk (*), 0.0021 with two asterisks (**), 0.0002 with three asterisks (***), and less than 0.0001 with four asterisks (****). A hash mark (#) indicates categories compared to the control, and an asterisk indicates categories compared to the previous category directly before it.

Most assays for autophagy modulators use the autophagy marker protein LC3B as the readout for autophagic activity. LC3B is a mammalian homolog of the yeast ATG8 protein, a ubiquitin-like protein that becomes lipidated and tightly associated with the autophagosomal membranes [19]. Autophagy levels were therefore measured by LC3B detection, a key marker for the autophagosome membrane structure. Increased levels of autophagy were observed in non-vaccinated individuals (Figure 7A). L-GSH treatment enhanced the levels of autophagy in BCG-vaccinated subjects (Figure 7B). In the granulomas of non-vaccinated subjects treated with PZA in the presence or absence of L-GSH, there was a significant increase of LC3B levels in all categories treated with L-GSH compared to the control and the categories without L-GSH treatment (Figure 7C). The same trend was found in the BCG-vaccinated subjects, with the exception of 1/10 PZA + L-GSH, with the only significant increase found when compared to the sham-treated group (Figure 7D). When comparing the non-vaccinated and BCG-vaccinated individuals directly, the BCG-vaccinated individuals showed a significant decrease in autophagy when compared to non-vaccinated individuals (Figure 7E). In response to PZA treatment with L-GSH, the LC3B levels were higher in the non-vaccinated group than the BCG-vaccinated group (Figure 7F). This may be a cell death mechanism to compensate for the low CD4 and high PD-1 in non-vaccinated subjects. In INH-treated granulomas, the presence of L-GSH lead to a significant increase in LC3B levels in the non-vaccinated group when compared to the sham-treated and the lone antibiotic-treated groups (Figure 7G). In BCG-vaccinated individuals, the MIC standalone INH showed a significant increase in autophagy compared to the control, and the addition of L-GSH to this category lead to a significant increase compared to the sham-treated and MIC standalone-treated groups (Figure 7H). When compared to the non-vaccinated group, there is less expression of LC3B in BCG-vaccinated individuals (Figure 7I). L-GSH treatment of granulomas from BCG-vaccinated subjects resulted in decreased expression of LC3B.

## 4. Discussion

Using *in vitro* granulomas derived from PBMCs, this study compares the differences in the immune responses against *M. tb* in both BCG-vaccinated and non-vaccinated subjects. The effects of GSH enhancement and the first line antibiotics (INH and PZA) in further enhancing the ability of immune cells to control *M. tb* infection in both the groups was also evaluated. This study also focuses on the mechanistic actions of PBMC-derived *in vitro* granuloma-like structures infected with the Erdman strain of *M. tb* from non-vaccinated subjects and BCG-vaccinated subjects to illustrate their immune response in a regulated environment. Our *in vitro* granulomas constituted cell types, such as macrophages, monocytes, and CD4 and CD8 T cells, all of which contribute to the immune responses necessary for granuloma formation.

We observed an active replication of *M. tb* to in the sham-treated granulomas, from the non-vaccinated group (Figure 1A). Furthermore, the addition of L-GSH led to a significant reduction in the viability of *M. tb* (Figure 1A). This confirms previous findings that indicate that L-GSH possesses antimycobacterial and immune-enhancing abilities. Furthermore, H&E staining was performed on these granuloma-like structures to visualize the morphology of these structures. The absence of L-GSH in the sham-treated category led to a formation of a sparse array of a granuloma, while L-GSH addition to this sample formed a larger, more robust granuloma-like structure (Figure 1A). This demonstrates the *M. tb* containment ability of L-GSH (Figure 1A). A similar trend was found in the BCG-vaccinated subjects. Notably, the mycobacterial count in the sham-treated control of the BCG-vaccinated group was 50% lower than the non-vaccinated subjects, and the addition of L-GSH lead to a further significant reduction in the bacterial load (Figure 1B). H&E staining demonstrated that the sham-treated and L-GSH-treated categories resulted in dense, robust granuloma-like structures, indicating the ability of BCG-vaccinated individuals to mount a robust immune response to hinder *M. tb* growth.

Findings from our study indicate that treatment of *in vitro* granulomas with PZA + L-GSH and INH+L-GSH resulted in a significant reduction in the bacterial load in the BCG-vaccinated samples compared to the non-vaccinated samples (Figure 1F,I). This evidence suggests that BCG vaccination enhances the ability of immune cells to effectively respond to GSH, PZA, and INH treatments, leading to containment and killing of *M. tb* (Figure 1E,F,I).

Our results also indicate that BCG-vaccinated individuals innately had higher levels of GSH when compared to the non-vaccinated group. Furthermore, L-GSH treatment resulted in a significant three log increase in the levels of GSH in BCG-vaccinated subjects when compared to non-vaccinated subjects (Figure 2A). PZA or INH treatments resulted in a significant increase in the levels of GSH in the granulomas from the BCG-vaccinated group when compared to the non-vaccinated group (Figure 2). Increased levels of GSH in the *in vitro* granulomas of BCG-vaccinated subjects further illustrate GSH’s antimycobacterial activity, as indicated by low *M. tb* numbers in the CFU assays in the BCG-vaccinated group in comparison to the non-vaccinated group.

Macrophages, CD4 and CD8 T cells play an important role in the control of *M. tb* infection. Cytokines, such as TNF-α, interleukin-2 (IL-2), and IFN-γ, produced by macrophages and T cells can enhance the effector responses against *M. tb* infection. In order to understand the immune effector mechanisms, by which BCG-vaccinated versus non-vaccinated subjects contain *M. tb* infection, we first measured the expression levels of CD4 in the granulomas. We initially observed the mean fluorescence of CD4 in the granulomas cultured in the presence and/or absence of L-GSH, PZA and PZA. We observed a dramatic, significant increase in the expression of CD4 in the granulomas from the BCG-vaccinated group with L-GSH addition (Figure 3E), PZA addition at every concentration of the antibiotic in the presence and absence of L-GSH, and INH + L-GSH treatment at every concentration of the antibiotic (Figure 3F,I), indicating the efficacy of L-GSH, PZA, and INH to maintain the viability CD4 T cells (Figure 3). This evidence also demonstrates that there is less CD4 T cell exhaustion in BCG-vaccinated subjects.

CD8^+^ T cells have also been shown to produce IFN-γ that can augment effector functions to control *M. tb* infection [20,21]. Importantly, CD8^+^ T cells possess cytolytic and cytotoxic functions to kill *M. tb* infected cells via a granule-mediated function (using perforin, granzymes, and granulysin). CD8 expression was determined in the granulomas of non-vaccinated and BCG-vaccinated subjects. Higher CD8 expression was observed following L-GSH treatment in the granulomas from both non-vaccinated and BCG-vaccinated subjects (Figure 4E). The addition of the PZA and INH in the presence or absence of L-GSH resulted in an increase in the expression of CD8 in the BCG-vaccinated category when compared to the non-vaccinated group (Figure 4F,I). Based on this general upward trend in the expression of CD8 in BCG-vaccinated subjects, we conclude that individuals who have been vaccinated with BCG have not only higher CD4 T cells in the presence of L-GSH, but also elevated levels of CD8 T cells.

A programmed death receptor 1 (PD-1) is a type I transmembrane protein expressed in immune cells, such as T, B, and NK cells [22,23]. When it binds to its receptor, PD-L1, PD-1 strongly interferes with T cell receptor (TCR) signal transduction [22,23]. PD-1 is a negative regulator of activated T cells, and blockage of this path restores T cell functions [10,24]. For this reason, our lab quantified the levels of PD-1 in non-vaccinated and BCG-vaccinated subjects. We observed a significant decrease in the expression of PD1 in the sham control, PZA-treated, and INH-treated granulomas from BCG-vaccinated individuals when compared to the same categories in the non-vaccinated group (Figure 5F,I). The addition of L-GSH significantly reduced PD-1 expression in both non-vaccinated and BCG-vaccinated groups (Figure 5A,B). These results indicate that low PD-1 expression in BCG-vaccinated individuals leads to less T cell exhaustion during *M. tb* infection. Decreased PD-1 expression in BCG-vaccinated subjects, along with increased CD4 and CD8 expressions, resulted in improved killing and containment of *M. tb*.

Cytokine production is a key determinate in the containment of a mycobacterial infection. TNF-α released during initiation of host immune response against *M. tb* infection causes recruitment of immune cells to form a granuloma while IFN-γ enhances effector functions of macrophages to control an intracellular *M. tb* infection. While TNF-α is a vital cytokine in containing *M. tb*, overexpression of TNF-α has been connected to multiple autoimmune inflammatory diseases [24,25,26]. Hence modulation of this cytokine is necessary. BCG-vaccinated individuals had higher levels of TNF-α in the sham-treated category (Figure 6A). L-GSH has a modulatory effect on the production of TNF-α in the BCG-vaccinated subjects (Figure 6A). Treatment of granulomas from BCG-vaccinated subjects with lower concentrations of PZA and INH in the presence or absence of L-GSH led to higher TNF-α production (Figure 6C,E). In the MIC concentration for both PZA and INH, TNF-α levels were diminished in the BCG-vaccinated categories. This is because at MIC, the CFUs were undetectable and, hence, there is restoration of homeostasis in the immune responses. L-GSH, PZA, and INH treatments also significantly increased the production of IFN-γ in granulomas from BCG-vaccinated subjects when compared to the non-vaccinated subjects (Figure 6D,F). This increased production of IFN-γ may be the mechanism by which BCG-vaccinated subjects can control *M. tb* infection.

Recently, autophagy has also been recognized as an immune effector mechanism against intracellular pathogens [27,28]. We observed that BCG-vaccinated individuals have a significant decrease in the expression of LC3B when compared to non-vaccinated individuals in the presence and absence of L-GSH, PZA and INH (Figure 7F,I). Our results indicate that autophagy could be a compensatory mechanism by which non-vaccinated individuals combat an *M. tb* infection.

Our study findings illustrate that the addition of L-GSH to the antibiotic treatment elicited a significant improvement in the granulomatous responses against *M. tb* infection. Our results indicate that in non-vaccinated individuals there was increased survival of *M. tb*, low CD8 T cell counts, and higher expression of PD-1. The addition of L-GSH to granulomas from non-vaccinated individuals restored the viability of CD8 and CD4 T cells and promoted autophagy. In contrast to the non-vaccinated subjects, BCG-vaccinated subjects innately able to kill *M. tb* more effectively, and this was accompanied by increased levels of GSH, higher CD8 counts, and decreased expression of PD-1. The addition of L-GSH to granulomas from BCG-vaccinated subjects lead to increased CD4 T cell viability, increased TNF-α and IFN-γ production, decreased PD-1 expression, and diminished T cell exhaustion. Therefore, we believe that enhancing GSH by means of L-GSH supplementation, along with anti-TB treatment, would not only reduce toxicity and treatment duration, but can also enhance the host immune responses to combat an active infection to promote enhanced treatment compliance.
